# Mutation-Driven Divergence and Convergence Indicate Adaptive Evolution of the Intracellular Human-Restricted Pathogen, *Bartonella bacilliformis*

**DOI:** 10.1371/journal.pntd.0004712

**Published:** 2016-05-11

**Authors:** Sandip Paul, Michael F. Minnick, Sujay Chattopadhyay

**Affiliations:** 1 Department of Microbiology, University of Washington, Seattle, Washington, United States of America; 2 Division of Biological Sciences, University of Montana, Missoula, Montana, United States of America; Beijing Institute of Microbiology and Epidemiology, CHINA

## Abstract

Among all species of *Bartonella*, human-restricted *Bartonella bacilliformis* is the most virulent but harbors one of the most reduced genomes. Carrión’s disease, the infection caused by *B*. *bacilliformis*, has been afflicting poor rural populations for centuries in the high-altitude valleys of the South American Andes, where the pathogen’s distribution is probably restricted by its sand fly vector’s range. Importantly, Carrión’s disease satisfies the criteria set by the World Health Organization for a disease amenable to elimination. However, to date, there are no genome-level studies to identify potential footprints of *B*. *bacilliformis* (patho)adaptation. Our comparative genomic approach demonstrates that the evolution of this intracellular pathogen is shaped predominantly via mutation. Analysis of strains having publicly-available genomes shows high mutational divergence of core genes leading to multiple sub-species. We infer that the sub-speciation event might have happened recently where a possible adaptive divergence was accelerated by intermediate emergence of a mutator phenotype. Also, within a sub-species the pathogen shows *inter*-clonal adaptive evolution evidenced by non-neutral accumulation of convergent amino acid mutations. A total of 67 non-recombinant core genes (over-representing functional categories like DNA repair, glucose metabolic process, ATP-binding and ligase) were identified as candidates evolving via adaptive mutational convergence. Such convergence, both at the level of genes and their encoded functions, indicates evolution of *B*. *bacilliformis* clones along common adaptive routes, while there was little diversity within a single clone.

## Author Summary

How host-restriction, intracellularity and genome reduction interplay to exert or maintain virulence is poorly characterized. The fact that *B*. *bacilliformis* is the most pathogenic *Bartonella* and has a highly reduced genome makes it an attractive model to gain insights into (patho)adaptive evolution of intracellular pathogens. Also, *B*. *bacilliformis* is known to lack many virulence genes present in other *Bartonella*, indicating unique strategies of (patho)adaptation. Our study reveals a prevalent nature of mutational force in *B*. *bacilliformis* evolution with two distinct outcomes: (a) mutational *divergence* leading to sub-speciation, possibly recently, via accelerated accumulation and fixation of favorable mutations mediated by a mutator phenotype; and (b) mutational *convergence* between clones of a sub-species exhibiting shared functional trajectories of adaptive evolution. Our findings highlight positions accumulating adaptive mutations in candidate genes, offering future functional studies to elucidate *B*. *bacilliformis* virulence evolution, and of broad application to intracellular pathogens with a reduced gene repertoire.

## Introduction

Bartonellae can serve as powerful models to study the evolution of intracellular, Gram-negative bacteria transmitted through the bite of hematophagous arthropods. As a genus, *Bartonella* currently includes 31 host-adapted species with varying degrees of pathogenicity [1]. While human-restricted *Bartonella bacilliformis* represents the most virulent *Bartonella* species [2], with fatality rates as high as 88% in untreated cases [3, 4] and a distribution restricted to South America [5], other bartonellae have a much wider geographical range and show significantly lower virulence potential in their corresponding mammalian hosts (e.g., *Bartonella quintana* causes human trench fever while *Bartonella henselae* causes asymptomatic feline infections and human cat-scratch disease world-wide) [6]. In addition, while core genes of different *Bartonella* species are found to be mostly syntenic, indicating a host-integrated metabolism [7], dynamic genome evolution is reflected at the species level; ranging from substantial genome expansion in *Bartonella tribocorum*, via gene duplications as well as lateral acquisition of prophages and genomic islands, to extensive genome reduction in species like *Bartonella clarridgeiae* and *B*. *bacilliformis* (PATRIC; http://www.patricbrc.org/) [8].

Conserved attributes of pathogenesis during human bartonelloses include bacteremia, erythrocyte parasitism (hemotrophy), infection of vascular endothelial cells, and pathological angiogenesis. Interestingly, *B*. *bacilliformis* represents the sole ancestral lineage in phylogenetic reconstructions of the genus, and lacks a number of virulence factors that are common to other *Bartonella* species (e.g., the type IV secretion system and corresponding substrate effector proteins used to subvert host cells) [9–11]. This conspicuous disparity and the marked virulence of *B*. *bacilliformis* suggest that the bacterium employs infection strategies that are distinct from other bartonellae, however, its virulence factors and pathogenomics are relatively under-characterized.

Carrión’s disease, the name given to the entire spectrum of clinical manifestations during a *B*. *bacilliformis* infection, affects an endemic population of about 1.7 million people limited to a defined altitudinal zone (600 to 3,200 m) of the Andean mountain valleys of Peru, Colombia and Ecuador, with reports of over 10,000 cases annually (Peruvian Ministry of Health, http://www.minsa.gob.pe/). High-risk populations include children (< 5 years old) and recent immigrants to endemic areas, although relatively recent reports document the spread of the disease into larger, non-endemic areas (e.g., in the Andean highlands and the Amazonian region east of the Andean mountains) [5, 12–15].

Little progress has thus far been achieved in understanding the molecular basis for host adaptation, differential disease presentations (e.g., Oroya fever, verruga peruana and chronic bacteremia) and the evolutionary mechanisms underlying the population diversity of *B*. *bacilliformis*. Previous comparative genomic approaches detected a set of genes orthologous to potential virulence genes of other *Bartonella* species (e.g., *B*. *tribocorum*), implying a similar role in *B*. *bacilliformis* [16]. However, to date, no study has been undertaken to delineate evolutionary forces that could affect genome-wide variations and potential pathoadaptation in *B*. *bacilliformis*. In this study, we compared publicly-available genomes of *B*. *bacilliformis* strains to construct pan-genomic profiles in order to decipher the relative contributions of horizontal gene transfer, recombination and mutation in shaping the bacterium’s evolution. While this study confirms a suspected sub-species structure in *B*. *bacilliformis*, we found mutation to be the primary force in both sub-species divergence and adaptive convergence within sub-species.

## Materials and Methods

### Phylogenetic analysis of MLST genes

For a set of 53 total strains (including 13 completely sequenced genomes), the maximum-likelihood based phylogeny was reconstructed using concatenated internal fragments of 7 housekeeping genes–*bvrR*, *flaA*, *ftsZ*, *groEL*, *ribC*, *rnpB and rpoB*–used previously [17] for multilocus sequence typing (MLST). We used MEGA6 [18] to calculate the sub-species diversity of *B*. *bacilliformis* based on internal fragments of 4 housekeeping genes (*gltA*, *groEL*, *ribC* and *rpoB*) used for assessing the sub-species diversity of *Bartonella vinsonii* [19].

### Pan- and core-genomic profiling of protein-coding genes

Since only the genome of strain KC583 was annotated and the remaining 12 strains had draft genomes (with the number of contigs ranging from 4 to 20), we first used MAUVE [20] to order the contigs of each draft genome using the annotated KC583 genome as a reference. The protein-coding genes of these draft genomes were then annotated using a combination of the RAST annotation server [21] and our recently developed software, PanCoreGen [22]. Using PanCoreGen, we constructed the pan-genomic profile at different nucleotide sequence identity threshold values (75%, 80%, 85%, 90% and 95%) keeping a constant 95% threshold for gene length-coverage in the process of identifying orthologous genes. For phylogenomic reconstruction and selection analysis, we used stringent cut-off values (95%) for both sequence-identity and gene length-coverage to determine the set of core genes, thereby eliminating highly diverse genes and minimizing the influence of non-homologous recombination or gene-shuffling. The phylogenomic tree was generated by MEGA6 [18] based on maximum parsimony.

### Analysis of recombination, selection and mutational convergence of core genes

Potential genes affected by homologous recombination events were detected using PhiPack software [23] that included three recombination-detection statistics: pairwise homoplasy index (Phi), maximum *χ*^2^ (MaxChi), and neighbor similarity score (NSS). If *P* values for all of the 3 statistics were <0.1, the gene was designated as recombinant [24].

Using DnaSP [25], we performed a McDonald-Kreitman test [26] to detect any positive selection footprints during versus after the divergence of a sub-species. Phylogenetic analysis of each core gene and detection of convergent structural mutations were performed by TimeZone software [27].

### Detection of prophage regions

In all 13 genomes, prophage regions were identified using PHAST (http://phast.wishartlab.com/) Web Server [28] by uploading the GenBank-formatted files for each genome. We considered genes from all regions designated as “intact”, “incomplete” or “questionable” by PHAST as prophage genes in our analysis.

### Simulations

EvolveAGene 3 [29] was used for gene-by-gene simulation of mutations under neutrality. Ten rounds of simulation were performed per gene. In each round, a random tree topology (with every branch having equal probability of leading to a terminal node or to an internal node) was generated considering the allele of the KC583 strain as the root sequence. Mutation rate, average branch lengths and average selection on amino acid replacements (i.e., dN/dS) were estimated from the phylogeny of a real data set for the corresponding gene. Neutrality was achieved by setting a constant default modifier value of 1 for the selection over sequence, and also over all branches in the phylogenetic tree. No indels were allowed in simulated datasets, since real datasets of core genes analyzed did not have any indels.

### Functional enrichment analysis

Clustering of candidate genes based on protein functions was done by DAVID software [30]. For the analysis, classification stringency was set to ‘medium’. Clusters were chosen to minimize the redundancy of genes representing encoded proteins in each functional group. An enrichment score higher than 0.5 and a *P* value less than 0.05 were used to assign a cluster as enriched or overrepresented.

## Results and Discussion

### A high diversity of housekeeping genes suggests sub-species structure in *B*. *bacilliformis*

The phylogenetic relationship of 13 sequenced strains (S1 Fig) was established based on 7 housekeeping genes used previously for MLST analysis [17]. While there was an overall average pairwise nucleotide diversity (*π*) of 0.011±0.001, we determined Ver097 to be distinct from the other strains. The *π* value of 0.044±0.004 between Ver097 and CAR (closest relative of Ver097 in the sample set) was significantly higher (P<0.0001) than the average *π* value (0.005±0.0004) for the other 12 strains. Also, we detected no correlation between evolutionary relationships among the strains and their geographical locations of isolation.

**Fig 1:**
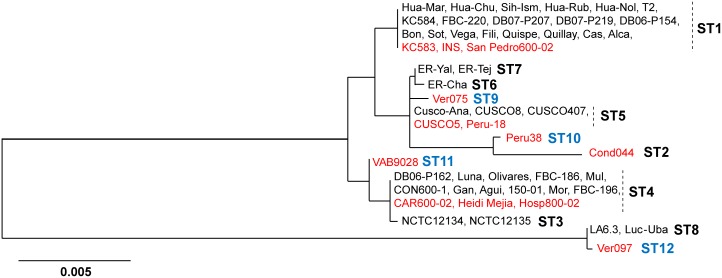
Phylogenetic reconstruction of *B*. *bacilliformis*. The tree was based on concatenated internal fragments of seven MLST loci (*bvrR*, *flaA*, *ftsZ*, *groEL*, *ribC*, *rnpB* and *rpoB*) from 13 completely sequenced strains (shown in red) and 43 strains analyzed in earlier work [17] (including three completely sequenced strains: KC583, CUCSO5 and Cond044).

Next, to assess the diversity of sequence types (ST), we combined our MLST dataset for 13 strains with that of 43 *B*. *bacilliformis* strains analyzed in earlier work [17], including strains KC583, CUSCO5 and Cond044 for which we had genome sequences in our analyzed set (Fig 1). We discovered 4 new ST groups represented by Ver075 (ST9), Peru38 (ST10), VAB9028 (ST11) and Ver097 (ST12). Interestingly, while previous work suggested that ST8 was a genospecies of *B*. *bacilliformis*, being well-distant from the rest [17], we found ST12 to have directly descended from ST8. In contrast, ST9, ST10 and ST11 remained closely-related to ST1 through ST7 with inter-ST nucleotide diversity (*π*) ranging from 0.0003 to 0.0102. Importantly, the *π* value between ST8 and ST12 was 0.0003±0.00033, i.e., identical to the lower limit of the diversity range among the other STs. This implied that ST8 and ST12 belong to a single clade well distant from the rest of the STs, thereby strengthening the possibility of multiple sub-species of *B*. *bacilliformis*.

To test this, we compared *B*. *bacilliformis* diversity with the sub-species diversity of *B*. *vinsonii* based on 4 housekeeping genes, as previously described [19]. No significant differences (P>0.10) were detected in the diversity values between Ver097 and rest of *B*. *bacilliformis* (*π* = 0.039±0.004) and the values between *B*. *vinsonii* sub-species (*arupensis-berkhofii π* = 0.053±0.005; *arupensis-vinsonii π* = 0.053±0.005; *berkhofii-vinsonii π* = 0.036±0.004). This strongly suggests that, similar to *B*. *vinsonii*, *B*. *bacilliformis* includes multiple sub-species. Combining information from Chaloner et al. [17], it appeared that, while ST1 through ST7 and ST9 through ST11 were members of one sub-species (referred to here as sub-species I), ST8 and ST12 joined to form another sub-species (designated as sub-species II) (Fig 1). ST8 strains (LA6.3 in 1990 and Luc-Uba in 1999) as well as ST12 representative Ver097 were isolated from the Ancash region of Peru, implicating this geographical area as a possible point of emergence for the sub-species. The MLST phylogeny of *B*. *bacilliformis* evolution using other *Bartonella* species as outgroups (S2 Fig) displayed deep branches coalescing to a common ancestor of the two sub-species. Such a phylogenetic structure suggests that the two *B*. *bacilliformis* sub-species might have diverged long ago from a common ancestor, rather than a recent emergence of sub-species II.

### Accessory genes reflect limited horizontal movements to and from *B*. *bacilliformis* genomes

**Fig 2:**
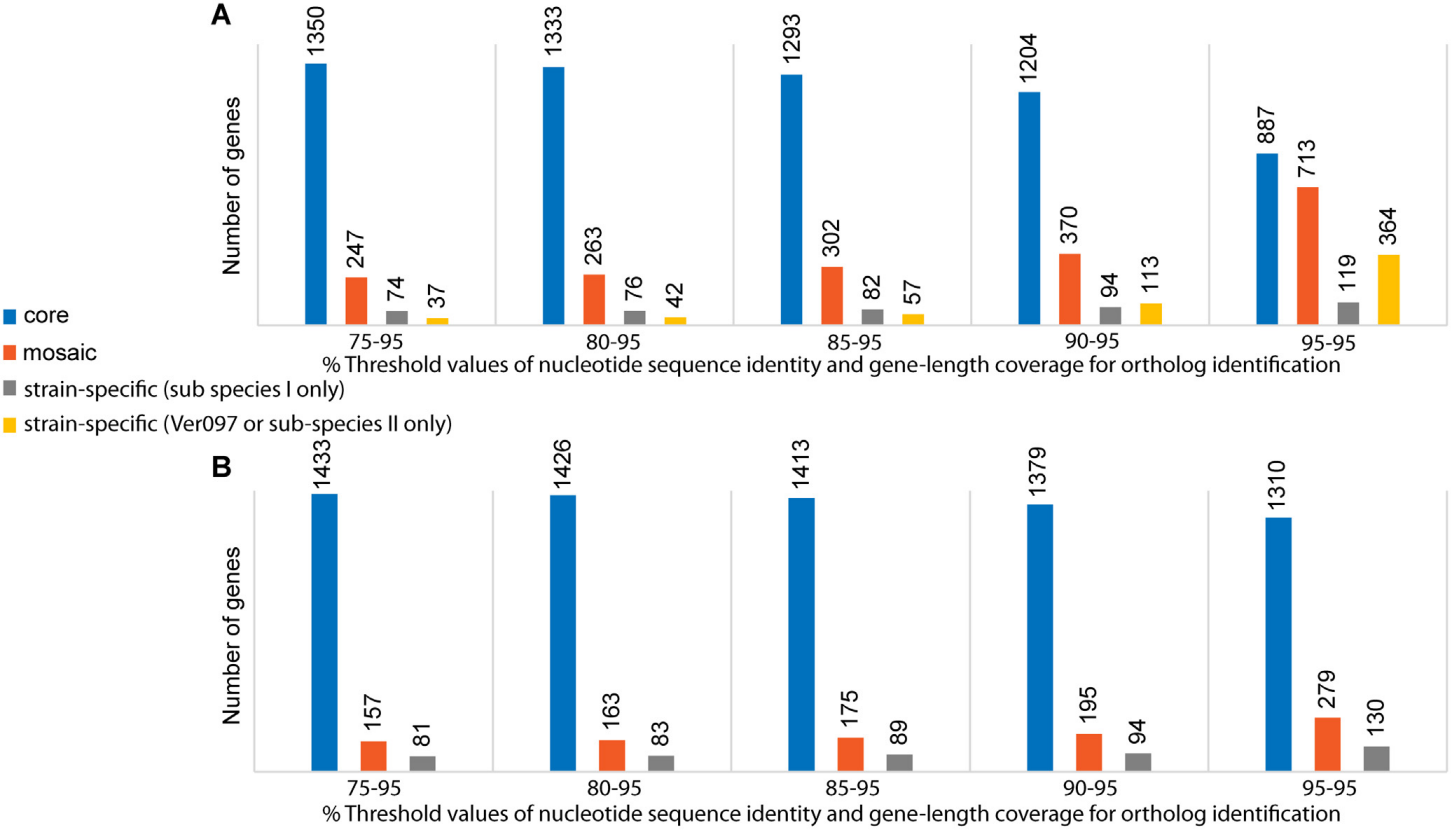
Pan-genomic distribution of core, mosaic and strain-specific genes. Analysis was performed among: (A) 13 strains, and (B) 12 strains (excluding Ver097) at different nucleotide sequence identity cut-offs while using a fixed 95% gene-length coverage cut-off for ortholog identification.

We next wanted to determine to what extent the MLST phylogeny would reflect the genomic scenario of 13 sequenced strains based on both gene content and diversity. We therefore performed a pan-genomic profiling of *B*. *bacilliformis*. In the first step, we chose a low, 75% cut-off value for nucleotide sequence identity while using a 95% threshold for gene-length coverage to identify orthologous genes. We found that 79% of the genes of the pan-genome were core, i.e., present in all 13 strains (Fig 2A). In the accessory fraction, 14% of genes were mosaic (i.e., present in multiple but not all strains) and 6% were strain-specific (i.e., unique to one of the 13 strains analyzed). Out of 74 strain-specific genes in sub-species I, 56 genes (76%) were harbored by Cond044, and 44 of these genes represented an intact prophage region of 36.3 kb (S1 Table). This suggested an increased frequency of horizontal gene transfer events in Cond044. While the prophage cluster in Cond044 was unique across all analyzed *B*. *bacilliformis*, a BLASTn search detected a short fragment (~7.5 kb) of this region in *B*. *vinsonii* subsp. *berkhoffii* at an 82% nucleotide identity level. Interestingly, 82% of this Cond044-specific region was also found in *B*. *tribocorum* (strains BM1374166 and CIP 105476) in different-sized fragments, each with multiple copies, ranging from 193 bp to 10.6 kb long (at 77–82% identity levels) and distributed throughout the genome. This supports the previous explanation of genome expansion in *B*. *tribocorum* via horizontal gene transfer and duplication events [8].

In contrast to Cond044, Ver097 harbored only 37 strain-specific genes (of which 65% were annotated as hypothetical), even though Ver097 showed up as an outlier based on the diversity of housekeeping genes (Fig 1). Therefore, it was evident that in separating Ver097 from the remaining 12 strains, unique gene content was not a major contributor, suggesting a limited role for horizontal transfer events in sub-speciation of *B*. *bacilliformis*. As a result, we hypothesized that high nucleotide diversity might have been acquired in an otherwise same set of genes, which ultimately led to the sub-species structure. To test this, we re-analyzed the pan-genomic profiling of *B*. *bacilliformis* at higher nucleotide identity cut-offs using the same 95% threshold for gene-length coverage (Fig 2A). We found a drastic drop in core genes to 43% and a rise in Ver097-specific genes (364 genes) at 95–95 cut-offs (i.e., 95% threshold levels for both nucleotide sequence identity and gene-length coverage). Importantly, the genomic locations of 327 Ver097-specific genes (i.e., the difference between Ver097-specific genes in 95–95 and 75–95 cut-offs) were distributed throughout the genome and were not limited to a few specific gene clusters (S3 Fig). This observation suggests accumulation of high nucleotide diversity in genes over the course of sub-species divergence, ruling out any significant contribution of gene mosaicism through horizontal gene transfer to sub-speciation.

The pan-genomic profile of *B*. *bacilliformis*, excluding Ver097, showed little variation in core, mosaic and strain-specific fractions at different identity thresholds (Fig 2B). For example, we detected only a 10% decrease in the core fraction as we increased cut-off values from 75–95 (86% core genes) to 95–95 (76% core genes), as opposed to a 36% decrease in core genes for the pan-genome based on all 13 genomes. Such a low fraction of accessory genes (14% using 75–95 cut-offs) suggested a limited contribution by horizontal transfer events, most of which affected a single strain, Cond044, as described above.

On the other hand, the core fraction comprised more than 75% of the genome. To gain an understanding of how sequence diversity impacted the phylogenomic relationship of the 13 strains, we reconstructed a phylogeny based on core genes. For orthologous gene identification, we used high thresholds (95–95) for nucleotide sequence identity and gene-length coverage, respectively, to essentially exclude highly-diverse genes resulting from non-homologous recombination or gene-shuffling. We identified 862 core genes that were then tested for the possible presence of homologous recombination. We identified 25 core genes as recombinants and excluded them from further analysis (S2 Table). We also excluded a total of 33 prophage genes (S3 Table) along with 50 annotated/un-annotated pseudogenes with truncation mutations (S4 Table) from the set of core genes. Finally, a *B*. *bacilliformis* phylogenomic tree was reconstructed using 754 core genes of possible mutational origin (S4 Fig). While Ver097, similar to the MLST tree (Fig 1), remained distant from the rest and re-confirmed the sub-species structure, we detected the presence of 4 distinct clades in the remaining 12 strains: (clade 1) KC583, INS, San Pedro600-02; (clade 2) Peru38, Ver075, Peru-18, CUSCO5; (clade 3) VAB9028, Hosp800-02, CAR600-02, Heidi Mejia; and (clade 4) Cond044. The within-clade diversity (*π*) ranged from 0.0002±0.00007 in clade 1 to 0.029±0.0005 in clade 2. However, between-clade diversity (*π*) was significantly higher (P<0.001), ranging from 0.034±0.001 (between clades 1 and 2) to 0.129±0.002 (between clades 2 and 4).

Altogether, results of MLST diversity, pan-genomic profiling and phylogenomic structure confirmed the existence of two sub-species of *B*. *bacilliformis*, where sub-species I maintained a tight clonal structure among 12 strains. Extrapolating the tightness of ST8 and ST12 in MLST phylogeny (Fig 1), we anticipate that sub-species II will show a similar picture. Also, we demonstrate that the emergence of new sub-species primarily resulted from enormous nucleotide divergence in common genes, rather than by horizontal transfer of phage genes and non-phage clusters of non-*B*. *bacilliformis* origin. The presence of a small number (3% of core genes) of recombinant genes suggests that nucleotide diversity predominantly arose via mutations in the core genomic fraction.

### Mutations converge within and between sub-species

In the set of 754 non-recombinant core genes, we wanted to assess the footprints of positive selection within and between sub-species. For this, we utilized evolutionary convergence of structural mutations, i.e., repeated independent (phylogenetically unlinked) mutations at the same amino acid positions of the encoded proteins. Positively-selected genes, i.e., (patho-)adaptive mutations that modify or inactivate functions of the encoded (virulence) proteins resulting in better fitness to the organism, are likely to be repeated when strains compete for survival under similar environmental conditions [31, 32]. Therefore, non-random accumulation of convergent structural mutations represents strong footprints of positive selection [33–37], especially in highly clonal populations as we determined here for *B*. *bacilliformis*. We detected a set of 152 genes (20% of the non-recombinant core fraction) that acquired convergent structural mutations. There were 61 (40%) genes that showed mutational convergence restricted to only the strains of sub-species I. In contrast, the remaining 91 (60%) genes were found to share at least one position that accumulated convergent mutations in Ver097 (the sole sub-species II representative) and the strains from sub-species I.

**Fig 3:**
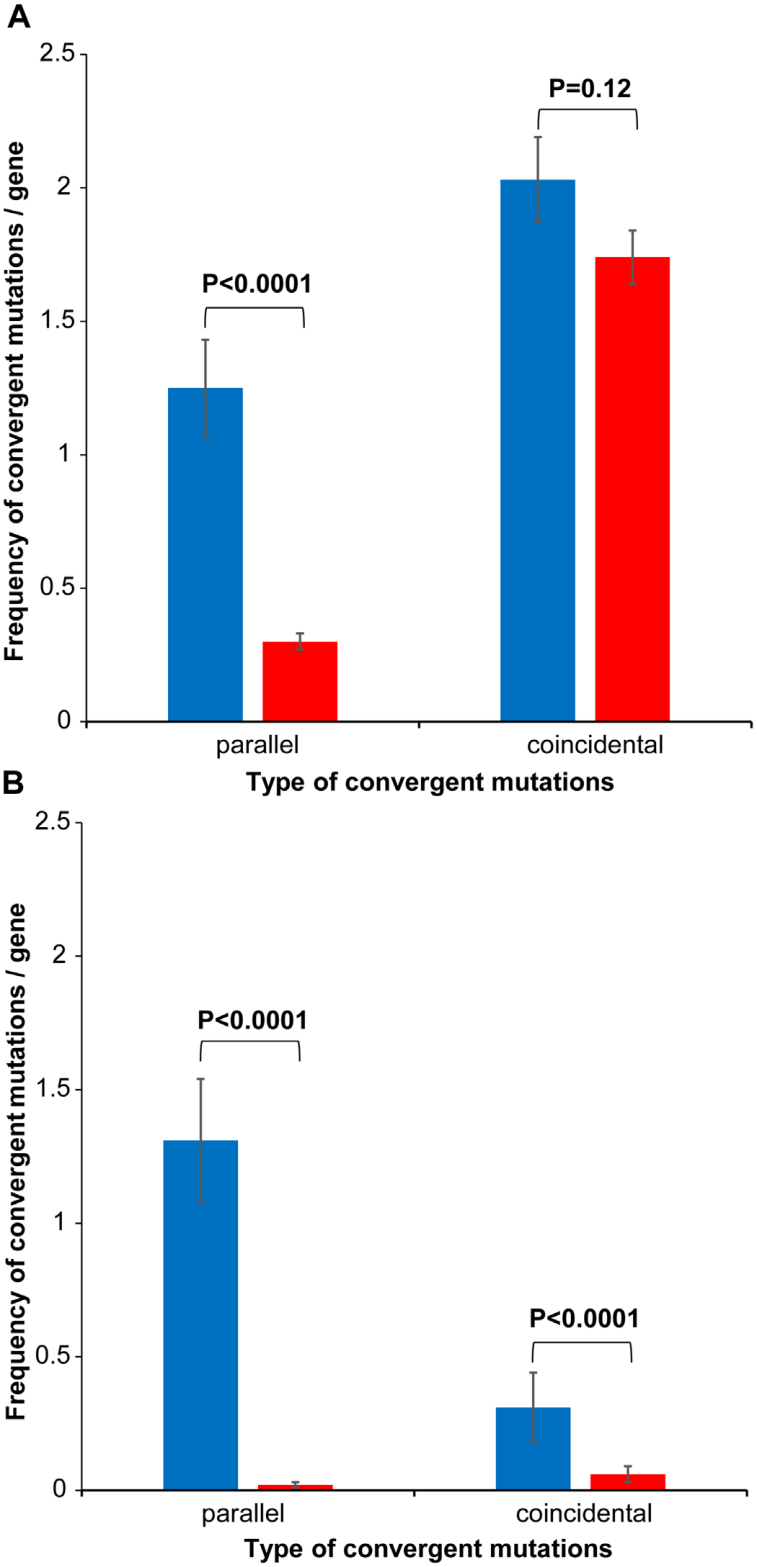
Frequency of parallel and coincidental convergent mutations in real (blue) and simulated (red) datasets. (A) 91 genes sharing convergent mutations between two sub-species (i.e., between Ver097 and one or more of the remaining 12 strains), and (B) 67 genes sharing convergent mutations within subspecies I (i.e., 12 strains excluding Ver097). Z-test P values are shown for each comparison between mean frequencies for real and simulated datasets.

Convergent mutations can be of two types, including parallel (identical mutations at the same amino acid positions) and coincidental (different mutations at the same amino acid positions). Surprisingly, we detected distinct features of convergence between sub-species in the set of 91 genes, compared to convergence within sub-species I in the set of 61 genes (Fig 3). The frequency of parallel convergent mutations (1.84±0.19) was ~2.7 times higher (P<0.0001) than coincidental mutations (0.69±0.14) in genes of the within sub-species set. In contrast, in genes of the between sub-species set, the frequency of coincidental mutations (2.03±0.16) was significantly higher (P = 0.0012) than parallel mutations (1.25±0.18). Also, there were significant differences in frequencies of parallel (P = 0.0244) and coincidental mutations (P<0.0001) between the two sets. Interestingly, if mutations were allowed to accumulate under neutrality, the frequency of coincidental convergent mutations would always be much higher than the parallel ones simply by random chance. On the other hand, previous work [38, 39] demonstrated the predominance of parallel convergence under positive selection, possibly responding to the need for precisely-tuned functional modification of proteins. By the same token, different replacements at specific positions (coincidental convergent mutations) critical for functional or structural integrity of encoded proteins can lead to possible immune escape or protein inactivation under adaptive pressures.

While parallel convergent changes may result from recombination, the coincidental ones (resulting in different changes) are always mutational events. Although we detected less than 3% of genes potentially affected by recombination, it is possible that the presence of homologous recombination might have been overlooked owing to a lack of sensitivity by the tools used. At this point, based on the predominance of coincidental convergent mutations between sub-species, we can confirm a mutational, non-recombinational origin of high nucleotide diversity that separated the two sub-species.

### Simulations detect non-random mutational convergence among clades of sub-species I

Consistent with the MLST (Fig 1) and phylogenomic (S4 Fig) trees, we observed that Ver097, the only representative of sub-species II, remained widely distant from sub-species I strains in almost every core gene phylogeny. To assess the evolutionary forces leading to the high mutational diversity that gave rise to the sub-species structure of *B*. *bacilliformis*, we performed simulation of mutations under the null hypothesis of no selection to examine two contrasting possibilities. It could be that sub-species II (Ver097) emerged under positive selection pressures leading to coincidental convergent changes. Alternatively, the separation of Ver097 from the rest by a long branch incorporating a large number of substitutions caused some pressures towards random acquisition of coincidental changes. Indeed, for the set of 91 genes showing convergence between sub-species, we detected no significant difference (P = 0.12) between the frequency of coincidental mutations in simulated data (1.74±0.10) and that of the real dataset, while the parallel mutation frequency in simulated data (0.30±0.03) was significantly lower (P<0.0001) than that in the real dataset (Fig 3A). The absence of positive selection pressures in the divergence of two sub-species was also supported by a McDonald-Kreitman test [26] with a P value of 0.74.

The scenario of excess coincidental convergent changes, equivalent to the expected frequency under neutrality, during sub-species divergence suggests a different possibility for the way sub-speciation might have occurred. Emergence of sub-species II could be a recent evolutionary incident wherein the sub-species was derived directly from sub-species I via hypermutation. This is an alternative to the divergence of two sub-species long ago in the evolution of *B*. *bacilliformis*, as suggested by MLST topology (S2 Fig). The recent emergence could be attributed to a mutator phenotype via certain defects in the DNA repair system. It is known that a low mutation rate leads to high metabolic costs in non-recombining microbial populations, and selection pressures sporadically drive allelic variations in genetic systems that dictate the precision of DNA replication and repair, thereby leading to an increased rate of mutation [40–42]. Importantly, we detected that *recF*, encoding a DNA replication and repair protein, was inactivated in Ver097 via a premature stop codon (S4 Table). It is known that restoration of a non-mutator phenotype is one of the primary roles of the DNA repair machinery [43]. Also, inactivation of RecF in intracellular microbes was previously shown to be linked to the loss of recombinational gene conversion [44].

Interestingly, we found that in Ver097, mutations predominantly targeted selected, mutable sites in specific genes across the genome (as evidenced by a high frequency of coincidental convergence, even higher than the simulated frequency under neutrality). If a mutator strain was the basis for the emergence of sub-species II, on one hand, the close relationship between ST8 and ST12 (Fig 1) indicates a massive reduction in mutation rate at some point after the rise of the mutator phenotype. On the other hand, similar to Ver097, the isolation of LA6.3 and Luc-Uba happened during 1990–1999 from Oroya fever patients in the Ancash region of Peru [45], depicting their stability and circulation in an otherwise endemic region. Models of clonal populations suggest that favorable mutants can arise in non-mutator genetic backgrounds from a mutator genotype [46], which in essence would lead to the fixation of genotypes (that we currently see in the environment) acquiring adaptive mutations on a fast track via hypermutation [47–49]. Since a trade-off due to continuous accumulation of deleterious mutations always exists, a reduction in mutation rate can be explained by the state where the fitness cost due to deleterious mutations exceeds the fitness gain level in metabolic activities [41, 50, 51]. We therefore hypothesize that natural selection might have favored the rise of a mutator phenotype in *B*. *bacilliformis* sub-species II with an aim to gain metabolic fitness. However, a more comprehensive understanding of the emergence will not be possible until genomes of more strains representing sub-species II are sequenced.

On the other side, we extracted all 67 core genes (S5 Table) that accumulated convergent mutations shared by the strains of sub-species I: (i) 61 genes showing convergence exclusively within sub-species I, and (ii) 6 genes with convergent mutations both within and between sub-species I. In the set of 12 strains of sub-species I, we detected a significant (P = 0.0002) predominance of parallel convergence (1.31±0.23) over the coincidental type (0.31±0.13) (Fig 3B). The simulated values for the corresponding dataset were extremely low (P<0.0001), with parallel and coincidental mutation-frequencies of 0.02±0.01 and 0.06±0.03, respectively (Fig 3B). Such non-random acquisition of convergent mutations strongly suggests the presence of positive selection in the evolution of *B*. *bacilliformis* sub-species I.

Therefore, the convergent changes, both of parallel and coincidental types, acquired in the course of sub-species I evolution cannot be explained by neutrality. Also, we detected that the mutations were converged exclusively between clades and not within a clade (i.e., not between the tightly-linked strains). This suggests the possibility of common fitness goals of the four clades that could lead to convergent adaptive pressures in sub-species I.

### Mutational convergence targets distinct functional groups of proteins

**Fig 4:**
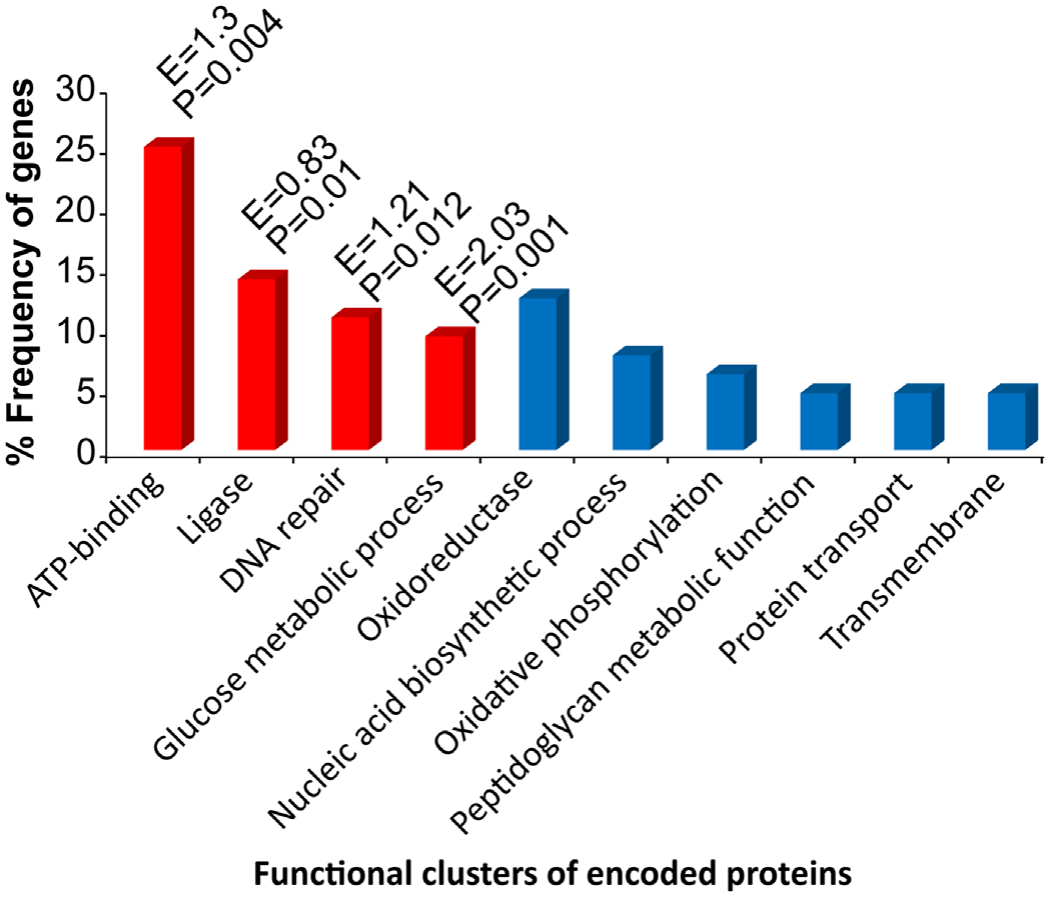
Functional clustering of encoded proteins for 67 candidate genes. These genes potentially evolved under positive selection for accumulating convergent mutations within subspecies I. Enrichment scores (E) and P-values are shown for the enriched or overrepresented functional clusters (in red).

Our next step was to determine whether the convergent adaptation we detected among clades in sub-species I was restricted to the level of genes targeted by convergent mutations, or whether adaptive convergence happened at the functional level of the encoded proteins. Functional enrichment analyses of 67 candidate genes (S5 Table) revealed the presence of 10 non-redundant functional clusters representing Gene Ontology (GO) categories of Biological Process, Molecular Function, and Domain (Fig 4). Interestingly, 4 of these clusters—DNA repair, glucose metabolic process, ATP-binding and ligase—were found to be enriched or overrepresented in our candidate gene-set.

Seven DNA repair genes encoding RecA, two DNA helicases (AddA, RuvA), a nuclease involved in lesion repair (UvrC), the endonuclease used to cleave the cruciform structure at the Holliday junction (RuvC), DNA ligase (LigA) and a base excision repair glycosylase (MutM) were identified as core genes under positive selection in sub-species I (S5 Table). The DNA repair functional cluster was previously shown to be under positive selection in ionizing-radiation-resistant-bacteria, a group renowned for its extraordinary ability to withstand both ionizing radiation and desiccation [52]. While *B*. *bacilliformis* is not normally exposed to extremes in water availability or ionizing radiation, the bacterium must nevertheless contend with disparate environments imposed by the sand fly vector’s midgut and the bloodstream of the human host. Moreover, as a facultative intracellular pathogen of humans, the bacterium would commonly encounter a variety of potentially DNA-damaging, immune-response effectors, such as reactive oxygen species. Thus, mutational convergence in genes representing this functional cluster would be clearly adaptive. Notably, experimental studies have identified *recA* as one of the pathogenicity genes in *B*. *tribocorum* [16].

Six genes involved in glucose metabolism were identified as candidates under positive selection. These included genes for phosphoglycerate kinase (*pgk*), transaldolase, glucose-6-phosphate isomerase (*pgi*), phosphopyruvate hydratase (*eno*), 2-oxoglutarate dehydrogenase (*sucA*), and glyceraldehyde-3-phosphate dehydrogenase (*gap*). These results suggest that the Emden-Meyerhof glycolytic pathway is undergoing “fine-tuning” in *B*. *bacilliformis* to accommodate disparate carbon sources and availability within the vector and/or human host cells. Indeed, altered regulation of carbon metabolism has previously been reported in strains of *Pseudomonas aeruginosa* isolated from cystic fibrosis patients, in support of this hypothesis [53].

The vast majority of genes identified as positively-selected in S5 Table are involved in metabolic processes. In addition to the six glucose metabolic genes described above, several genes involved in ATP synthesis (*atpG*, *atpD*), electron transport (*nuoM*, *nuoD*, BARBAKC583_0816), vitamin B synthesis (*cobT*, *folC*), amino acid metabolism (*proB*, *gcvP*, *lys1*, *aroK*) and nucleotide biosynthesis (*pyrG*, *guaA*, BARBAKC583_0178) were identified. These data suggest that metabolic pathways of *B*. *bacilliformis* are under considerable selective pressure, possibly reflecting the pathogen’s evolution towards optimizing its ability to reside and survive in both sand flies [54] and humans, where conditions are quite dissimilar. It is tempting to speculate that these genes may contribute to the enhanced virulence of *B*. *bacilliformis* relative to other pathogenic bartonellae. Interestingly, recent work examining pathoadaptive evolution of *Salmonella* Typhimurium during chronic infections of mice identified a known metabolic regulator (KdgR), where a single nucleotide polymorphism significantly enhanced bacterial transmission between littermates and colonization of the intestines relative to a wild-type parental strain [55].

Positively-selected genes involved those encoding five potential transporters, including an amino acid ABC transporter (BARBAKC583_0595), a glucan ABC transporter potentially involved in osmoregulation, a Bcr/Cfla subfamily drug resistance transporter (BARBAKC583_0866), a RND membrane fusion protein (MFP) family efflux transporter (BARBAKC583_0305) and a potential macrolide-specific efflux protein MacA (BARBAKC583_0096). In addition, the PhoU regulator for the phosphate transport system and a transporter facilitator (BARBAKC583_0895) were found to be positively-selected. Of particular interest are the potential drug efflux pumps, Bcr/Cfla, the RND MFP and MacA. Bcr/Clfa homologs have demonstrated resistance activity against bicyclomycin, chloramphenicol and florfenicol in various Gram-negative bacteria, while both RND and MacA homologs include transporters for bacterial hemolysins and drug efflux. Of possible relevance is that acute manifestations of Carrión’s disease (Oroya fever) have been treated for many decades with chloramphenicol, while macrolides have been used as second-line antimicrobials to treat both acute and chronic forms (verruga peruana) of disease [56]. Thus, selective pressure to acquire resistance to antimicrobials would be prevalent in endemic areas.

A handful of genes involved in translation were found to be under positive selection in *B*. *bacilliformis*, including the beta subunit of glycyl tRNA synthetase (*glyS*), two ribosomal proteins (*rplQ*, *rpsF*) and ribosome recycling factor (*frr*). Typically, evolution of translation-related genes is relatively slow, to ensure maintenance of structural and functional integrity of the essential gene products [57, 58]. Presumably, these genes are under strong selective pressure to maintain or optimize overall translational efficiency in *B*. *bacilliformis*.

The cell wall constitutes an essential protective barrier against the extracellular environment and an interface between the bacterial cell and its niche. Four genes were identified as positively-selected in this category. The first, *lolA* (BARBACK583_0094) encodes an outer membrane lipoprotein chaperone. The second gene, *mraY*, encodes a protein involved in the transfer of peptidoglycan precursors from the cytosol to bactoprenol for transport across the cell membrane. Third, *murD*, encodes a ligase for production of peptidoglycan precursors in the cytosol. Finally, a peptidoglycan recognition protein (PRP) amidase (BARBACK583_0904) was identified. Interestingly, no obvious membrane proteins with potential virulence function [56]–such as *Bartonella* repeat proteins (Brps), hemin-binding proteins (Hbps), flagellin (FlaA) and invasion-associated locus A (IalA)–were found to be under selection for accumulation of convergent mutations. Although reasons for this are unclear, it could be that immunological pressure for these proteins is minimal, despite their physical location in the cell and potential exposure to acquired and innate immune effectors. Alternatively, these membrane proteins provide essential virulence functions in the context of the sand fly vector and/or human host and are therefore indispensable to bacterial fitness and survival.

In *B*. *tribocorum*, signature-tagged mutagenesis studies detected 97 genes linked to pathogenesis, of which 66 genes had orthologs in *B*. *bacilliformis* [16]. Only four of these genes, including *recA* discussed above, were found to undergo adaptive evolution via mutational convergence. Three other genes included BARBAKC583_0153 (hypothetical), BARBAKC583_0335 (encoding sugar isomerase of KpsF/GutQ family) and BARBAKC583_1364 (hypothetical), although in *B*. *tribocorum* these genes were annotated as either putative regulators or signal peptide proteins. This insignificant overlap, along with complete absence of several virulence genes found in *B*. *tribocorum* and other *Bartonella* species, indicates markedly different pathoadaptation strategies for *B*. *bacilliformis*. Indeed, the highly reduced genome of *B*. *bacilliformis* relative to other species such as *B*. *tribocorum*, with its expanded genome arising from horizontal transfer and duplication events [8], underscores these differences.

With recent advances in high-throughput sequencing technologies, population genomics studies are warranted to associate genetic variation with a pathogen’s disease potential, and convergence-based association is a powerful approach [37, 59], especially in clonal populations of species like *B*. *bacilliformis*. Our study maps, for the first time, convergent adaptive evolution of protein-coding genes across the highly-reduced genome of a facultative intracellular pathogen. Candidate genes and the potential adaptive mutations therein, should serve as an important resource for functional studies leading to a better understanding of *B*. *bacilliformis* pathogenesis as well as possible common virulence characteristics of intracellular pathogens harboring reduced genomes. As we re-confirm the sub-species structure of *B*. *bacilliformis*, we hypothesize that the sub-speciation event might be evolutionarily recent—triggered by the emergence of a mutator strain, followed by the fixation of adaptive variants in a non-mutator background. However, the possibility that two sub-species diverged from a common ancestor cannot be ruled out. To decipher how, why and when sub-speciation happened, additional genome sequencing and more information regarding strains and clinical history of the patients from which they were isolated is crucial. We believe this study provides a stepping stone toward future translational research, such as clinical diagnostics, epidemiology, and environmental control of *B*. *bacilliformis*, thereby facilitating elimination of Carrión’s disease in poor, rural, mountain communities of South America.

## Supporting Information

S1 Fig Strain-information and phylogenetic reconstruction of 13 completely sequenced *B*. *bacilliformis* genomes. The tree was based on MLST using internal fragments of seven housekeeping genes (*bvrR*, *flaA*, *ftsZ*, *groEL*, *ribC*, *rnpB* and *rpoB*).
(TIF)

S2 Fig Phylogenetic reconstruction of 13 completely sequenced *B*. *bacilliformis* genomes along with other *Bartonella* species. The tree was based on internal fragments of five housekeeping genes (*batR*, *gltA*, *groEL*, *ribC* and *rpoB*).
(TIF)

S3 Fig Genomic locations of 327 Ver097-specific genes across the Ver097 constructed genome. These genes represented the set detected as specific to Ver097 using 95–95 cut-offs for % nucleotide identity and gene length-coverage, but not using 75–95 cut-offs (Fig 2).
(TIF)

S4 Fig Phylogenomic reconstruction of 754 core genes of mutational origin in 13 *B*. *bacilliformis* genomes. (TIF)

S1 Table RAST annotated genes in strain-specific prophage region of Cond044. This region represents position 779373 bp to 815728 bp of acOtG-supercont1.2.C7 supercontig in the whole genome shotgun sequence of Cond044.
(PDF)

S2 Table List of core recombinant genes. Gene annotations are based on the reference strain KC583.
(PDF)

S3 Table List of core phage genes predicted by PHAST. Gene annotations are based on the reference strain KC583.
(PDF)

S4 Table List of genes with truncation mutations in one or multiple strains. Gene annotations are based on the reference strain KC583.
(PDF)

S5 Table List of candidate non-recombinant core genes with adaptive convergent mutations in *B*. *bacilliformis* subspecies I. Gene annotations are based on reference strain KC583. Amino acid positions accumulating convergent mutations are shown. Genes representing enriched (or overrepresented) functional clusters are grouped.
(PDF)
