# Supplementary figures and images for: Mutation-Driven Divergence and Convergence Indicate Adaptive Evolution of the Intracellular Human-Restricted Pathogen, Bartonella bacilliformis
Source: PLoS Negl Trop Dis. 2016 May 11;10(5):e0004712. doi: 10.1371/journal.pntd.0004712 (PMC4864206; doi:10.1371/journal.pntd.0004712)

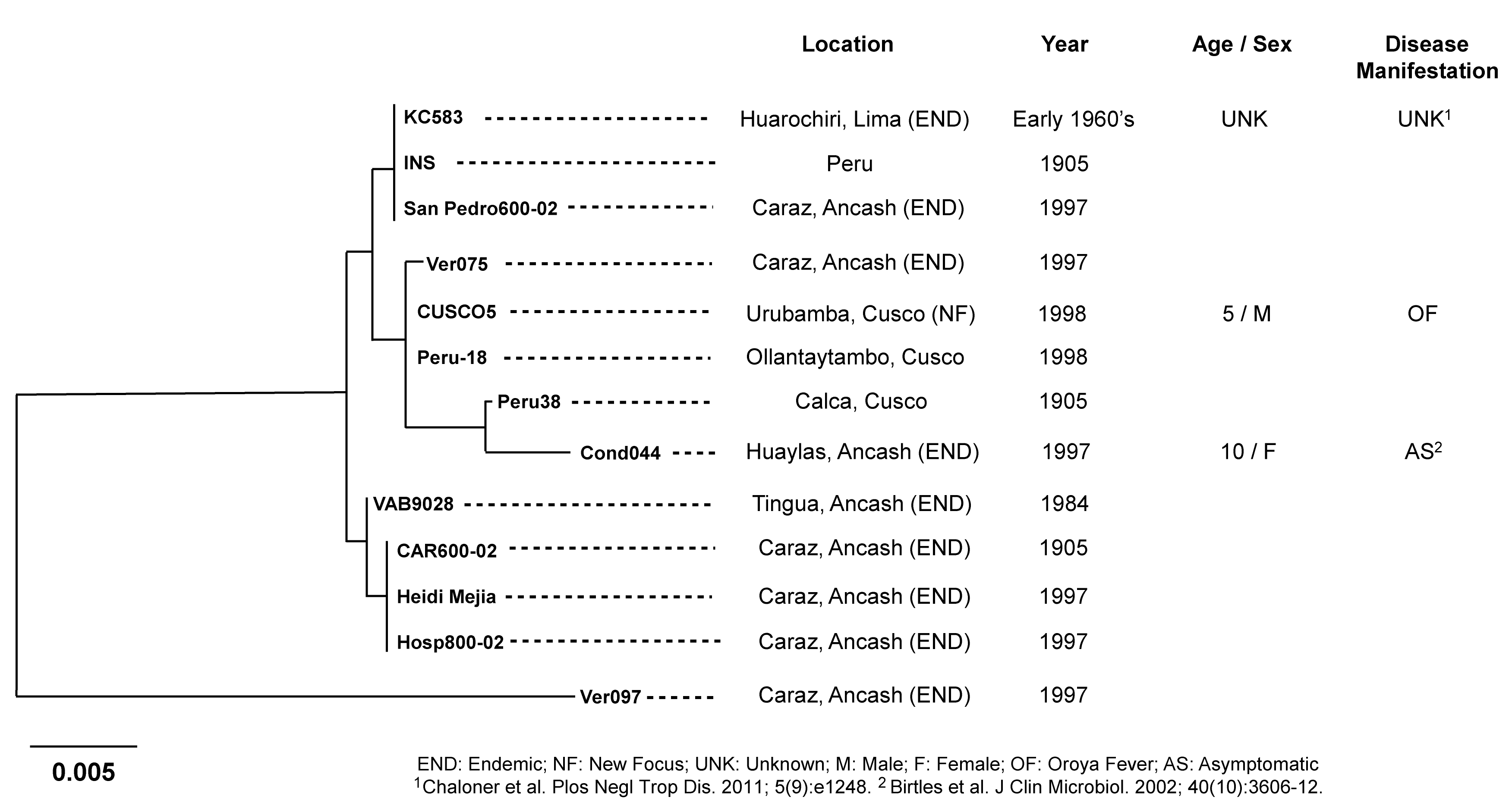

Supplement: S1 Fig — The tree was based on MLST using internal fragments of seven housekeeping genes (bvrR, flaA, ftsZ, groEL, ribC, rnpB and rpoB). (TIF) [file pntd.0004712.s001.tif]

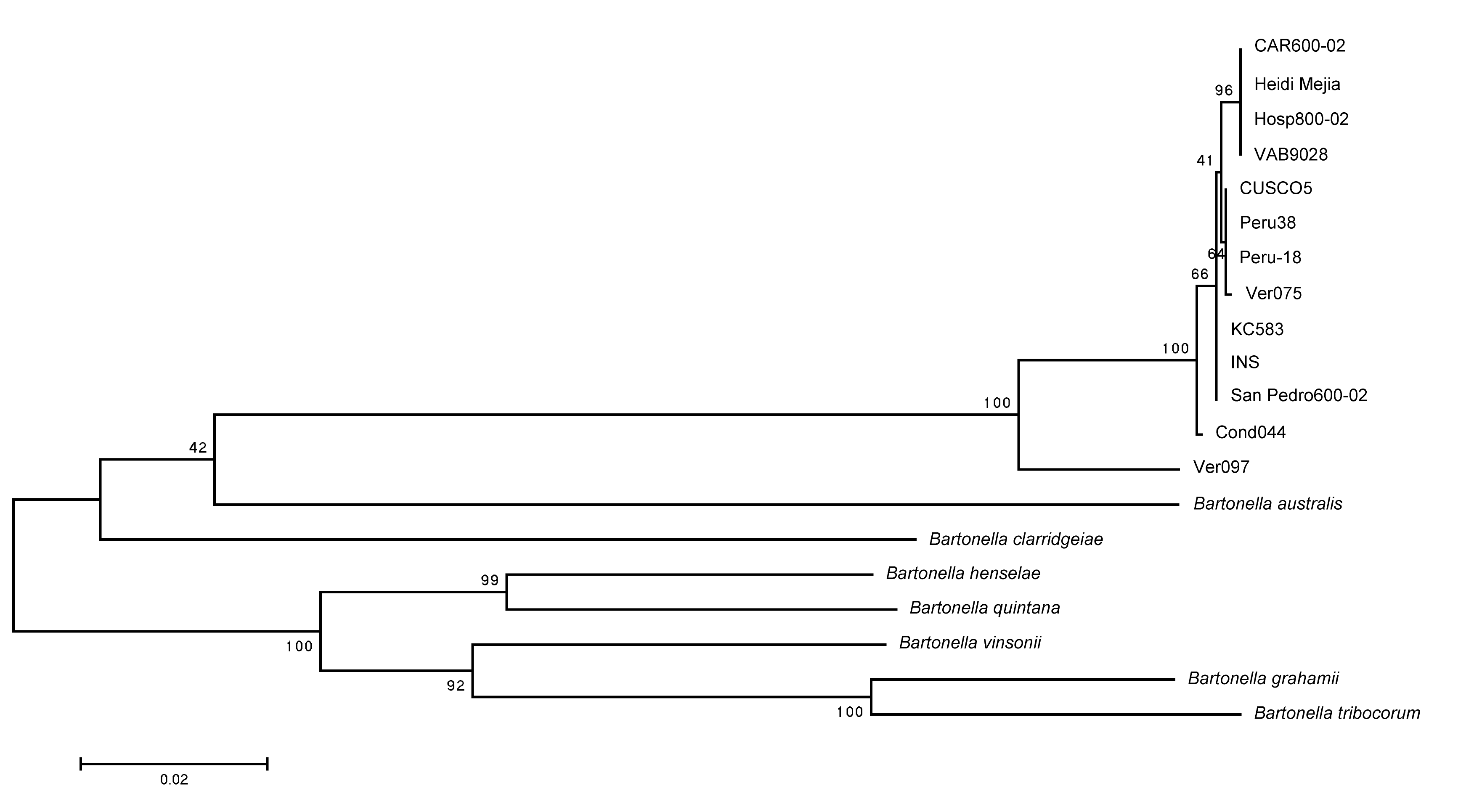

Supplement: S2 Fig — The tree was based on internal fragments of five housekeeping genes (batR, gltA, groEL, ribC and rpoB). (TIF) [file pntd.0004712.s002.tif]

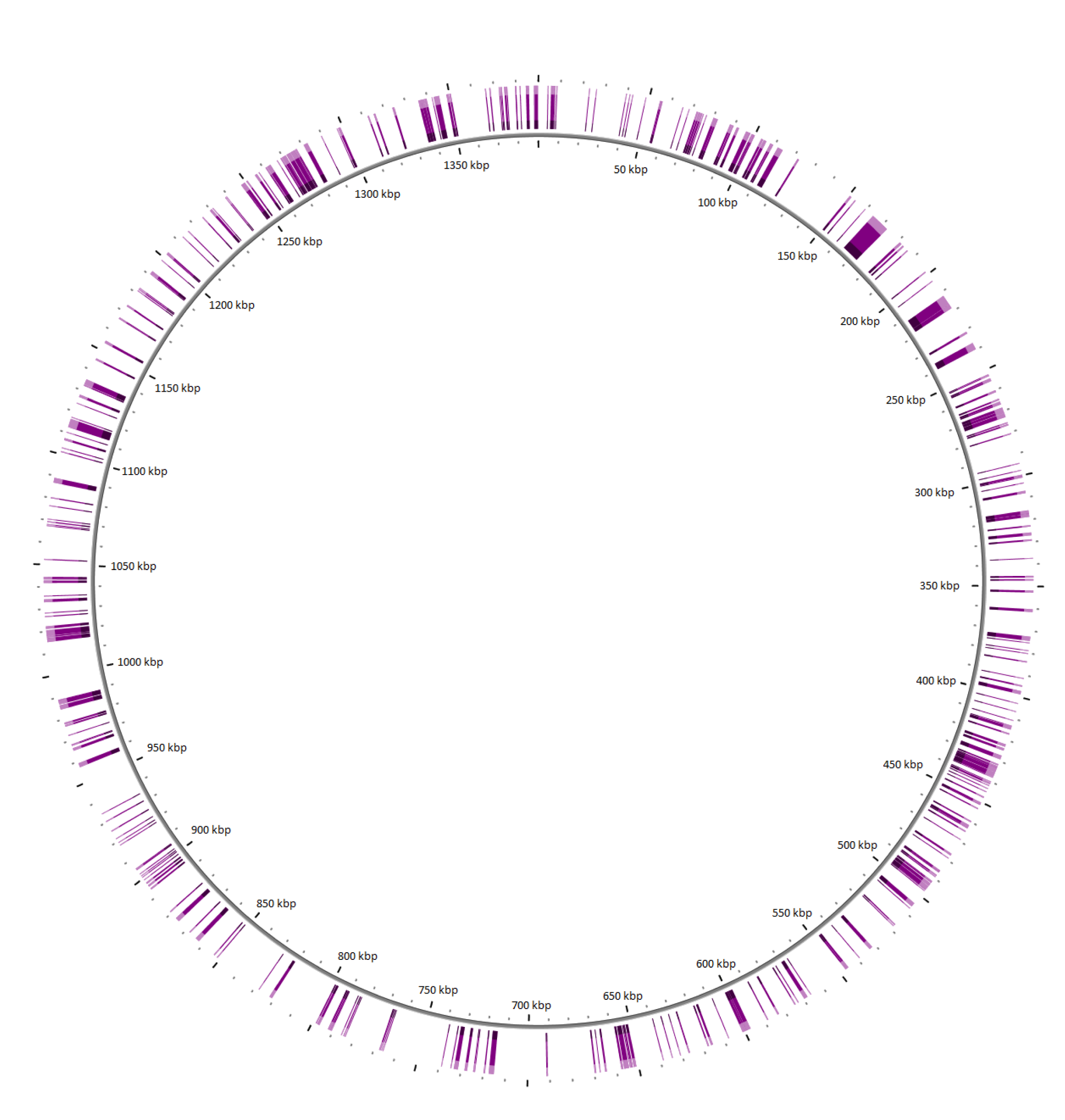

Supplement: S3 Fig — These genes represented the set detected as specific to Ver097 using 95–95 cut-offs for % nucleotide identity and gene length-coverage, but not using 75–95 cut-offs (Fig 2). (TIF) [file pntd.0004712.s003.tif]

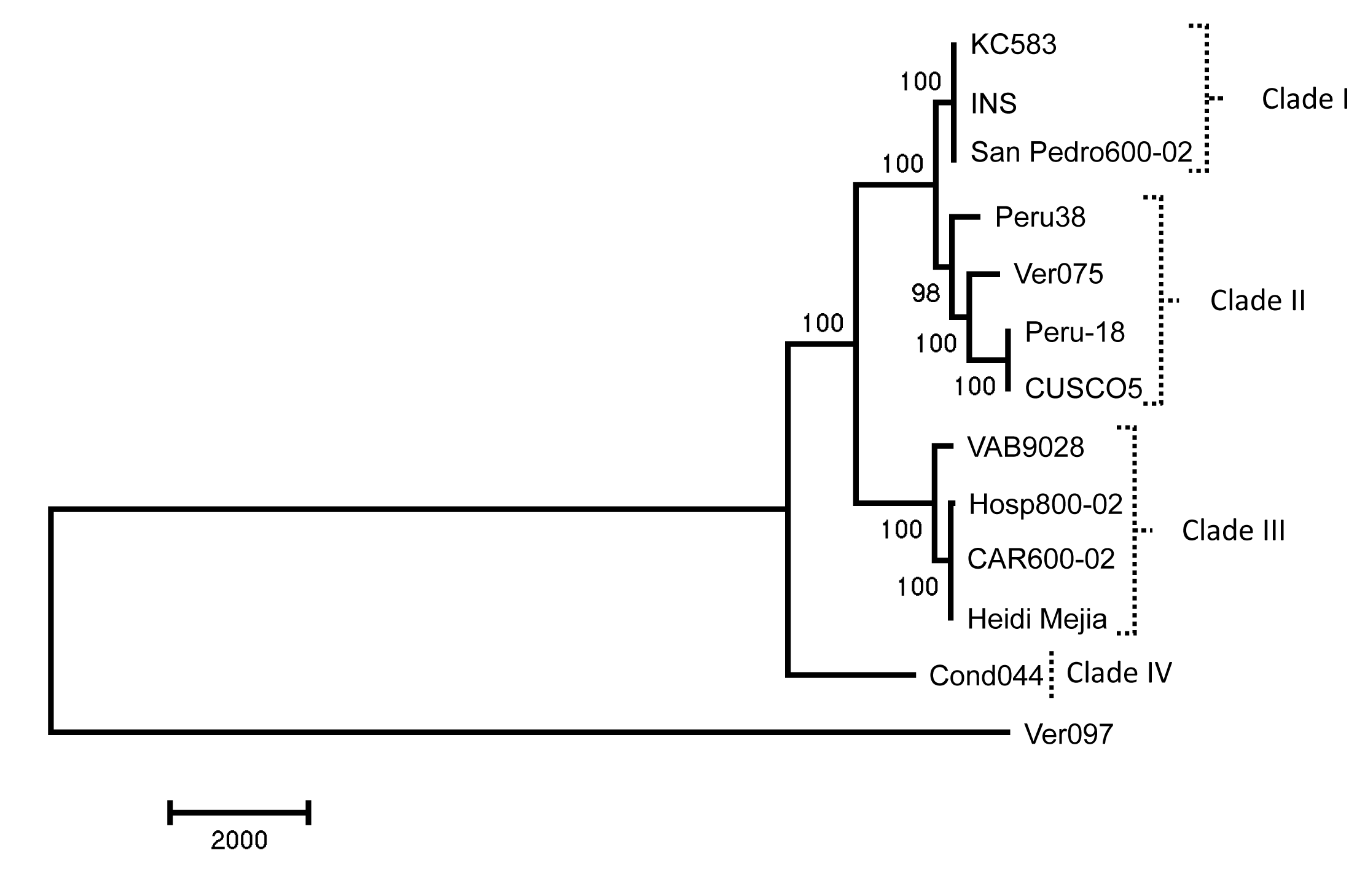

Supplement: S4 Fig — (TIF) [file pntd.0004712.s004.tif]
