# Supplementary material for: Mutation-Driven Divergence and Convergence Indicate Adaptive Evolution of the Intracellular Human-Restricted Pathogen, Bartonella bacilliformis
Source: PLoS Negl Trop Dis. 2016 May 11;10(5):e0004712. doi: 10.1371/journal.pntd.0004712 (PMC4864206; doi:10.1371/journal.pntd.0004712)
Supplement: S1 Table — This region represents position 779373 bp to 815728 bp of acOtG-supercont1.2.C7 supercontig in the whole genome shotgun sequence of Cond044. (PDF) [file pntd.0004712.s005.pdf]

S1 Table. RAST annotated genes in strain-specific prophage region of Cond044. This region represents position 779373 bp to 815728 bp of acOtG-supercont1.2.C7 supercontig in the whole genome shotgun sequence of Cond044.

Product	Protein length (AA)
Phage protein D	337
hypothetical protein	59
putative phage tail protein	138
Phage tail length tape-measure protein	760
hypothetical protein	137
Phage tail tube protein FII	172
Phage tail sheath monomer	427
hypothetical protein	71
PE-PGRS FAMILY PROTEIN	455
Virulence-associated protein	1047
phage protein	368
Phage-related baseplate assembly protein	282
phage-related baseplate assembly protein	128
hypothetical protein	60
gpV	197
hypothetical protein	176
Mu-like prophage protein gpG	164
Mu-like prophage protein gp36	140
hypothetical protein	321
hypothetical protein	113
Mu-like prophage I protein-like	335
Phage (Mu-like) virion morphogenesis protein	401
Mu-like prophage FluMu protein gp29	517
Phage terminase, large subunit	535
Mu-like prophage FluMu protein GP27	193
phage related protein	118
hypothetical protein	104
hypothetical protein	53
hypothetical protein	112
hypothetical protein	95
Membrane-bound lytic murein transglycosylase D precursor (EC 3.2.1.-)	195
hypothetical protein	135
Mu-like prophage protein gp16	205
Phage protein	207
hypothetical protein	92
hypothetical protein	90

Mobile element protein	343
Mobile element protein	660
hypothetical protein	152
Chromosome (plasmid) partitioning protein ParB	289
Chromosome (plasmid) partitioning protein ParB	136
hypothetical protein	97
Predicted transcriptional regulators	176
hypothetical protein	40
