# Supplementary material for: Mutation-Driven Divergence and Convergence Indicate Adaptive Evolution of the Intracellular Human-Restricted Pathogen, Bartonella bacilliformis
Source: PLoS Negl Trop Dis. 2016 May 11;10(5):e0004712. doi: 10.1371/journal.pntd.0004712 (PMC4864206; doi:10.1371/journal.pntd.0004712)
Supplement: S2 Table — Gene annotations are based on the reference strain KC583. (PDF) [file pntd.0004712.s006.pdf]

S2 Table. List of core recombinant genes. Gene annotations are based on the reference strain KC583.

Gene	GI	Strand	CDS-region	Product	Protein length (AA)
phaAB	120614568	+	35897-38818	pH adaptation potassium efflux system protein A/B	973
BARBAKC583_0057	120614466	+	60099-62516	conserved hypothetical protein TIGR02302	805
BARBAKC583_0072	120615084	+	80897-82267	putative ribosomal RNA small subunit methyltransferase B	456
sdhA	120614198	+	98370-100217	succinate dehydrogenase, flavoprotein subunit	615
BARBAKC583_0093	120614049	+	103019-105439	FtsK/SpoIIIE family protein	806
leuS	120615057	+	117102-119729	leucyl-tRNA synthetase	875
atpA	120614890	+	125733-127268	ATP synthase F1, alpha subunit	511
BARBAKC583_0131	120614370	+	145305-147134	ABC transporter, ATP-binding protein	609
glmM	120614848	+	182309-183661	phosphoglucosamine mutase	450
BARBAKC583_0228	120613933	+	226952-228838	conserved hypothetical protein	628
BARBAKC583_0238	120614093	+	237453-238424	putative phage protein	323
BARBAKC583_0291	120614955	+	276703-279321	FtsK/SpoIIIE family protein	872
BARBAKC583_0306	120614682	+	293733-296867	RND transporter, hydrophobe/amphiphile efflux-1 (HAE1) family	1044
BARBAKC583_0315	120614286	+	308017-308562	invasion associated locus B family protein	181
secA	120614914	-	355713-358445	preprotein translocase, SecA subunit	910
cycK	120614271	+	434255-436234	cytochrome c-type biogenesis protein CycK	659
pepN	120614263	-	443458-446085	aminopeptidase N	875
pcs	120615018	+	836940-837701	phosphatidylcholine synthase	253
BARBAKC583_0936	120614664	+	961496-963319	peptidase, M24 family	607
dnaG	120614328	-	999691-1001631	DNA primase	646
BARBAKC583_0969	120614834	+	1003044-1004732	Na+/Pi-cotransporter family protein	562
BARBAKC583_1113	120614789	+	1152018-1152968	iron chelate ABC transporter, permease protein	316
dapE	120614561	-	1392461-1393633	acetylornithine deacetylase/succinyl-diaminopimelate desuccinylase	390
BARBAKC583_1342	120614693	+	1396249-1398015	ABC transporter, permease/ATP-binding protein	588
unannotated gene in strain SanPedro600-02	RAST-annotated			ClpB protein	793
