# Supplementary material for: Mutation-Driven Divergence and Convergence Indicate Adaptive Evolution of the Intracellular Human-Restricted Pathogen, Bartonella bacilliformis
Source: PLoS Negl Trop Dis. 2016 May 11;10(5):e0004712. doi: 10.1371/journal.pntd.0004712 (PMC4864206; doi:10.1371/journal.pntd.0004712)
Supplement: S3 Table — Gene annotations are based on the reference strain KC583. (PDF) [file pntd.0004712.s007.pdf]

S3 Table. List of core phage genes predicted by PHAST. Gene annotations are based on the reference strain KC583.

Name	GI	Strand	CDS-region	Product	Protein length (AA)
BARBAKC583_0233	120614191	+	233396-234859	conserved hypothetical protein	487
BARBAKC583_0240	120614250	+	239158-239820	phage lysozyme	220
BARBAKC583_0894	120614929	+	920779-921966	conserved hypothetical protein	395
BARBAKC583_0895	120614363	+	921960-923168	transporter, major facilitator family	402
BARBAKC583_0896	120615070	+	923603-924247	LemA family protein	214
parE	120614743	+	926128-928206	DNA topoisomerase IV, B subunit	692
recJ	120613912	-	928224-930008	single-stranded-DNA-specific exonuclease RecJ	594
rplM	120614747	+	930296-930760	ribosomal protein L13	154
rpsI	120613988	+	930763-931248	ribosomal protein S9	161
divK	120614910	-	931408-931773	cell division response regulator DivK	121
BARBAKC583_0904	120614211	-	932336-933100	N-acetylmuramoyl-L-alanine amidase family protein	254
cycA	120614399	+	941025-942425	D-serine/D-alanine/glycine transporter	466
ilvC	120613911	-	942499-943518	ketol-acid reductoisomerase	339
BARBAKC583_0918	120613871	-	944210-944944	putative pyridoxal phosphate biosynthesis protein PdxJ	244
BARBAKC583_0919	120614472	-	945025-946134	conserved hypothetical protein	369
BARBAKC583_0922	120614871	-	947859-949349	protease Do family protein	496
BARBAKC583_0923	120614348	-	949572-950444	putative HflC protein	290
folA	120614711	-	951709-952218	dihydrofolate reductase	169
thyA	120614502	-	952215-953009	thymidylate synthase	264
kgtP	120614304	+	953222-954529	alpha-ketoglutarate permease	435
BARBAKC583_0930	120614923	+	955540-956073	conserved hypothetical protein	177
BARBAKC583_0933	120614621	-	959137-959766	bacterial transferase hexapeptide repeat protein	209
BARBAKC583_0934	120614540	+	959998-960576	SCO1/SenC family protein	192
prmA	120613934	+	960603-961478	ribosomal protein L11 methyltransferase	291
ligA	120614712	-	963536-965689	DNA ligase, NAD-dependent	717

recN	120614782	-	965805-967472	DNA repair protein RecN	555
BARBAKC583_0939	120614239	-	967479-968318	putative lipoprotein	279
BARBAKC583_1026	120614357	-	1061750-1062532	dipeptide ABC transporter, ATP-binding protein	260
BARBAKC583_1027	120614608	-	1062529-1063404	dipeptide ABC transporter, ATP-binding protein	291
unannotated gene in KC583	RAST-annotated			tRNA dimethylallyltransferase (EC 2.5.1.75)	77
unannotated gene in KC583	RAST-annotated			tRNA dimethylallyltransferase (EC 2.5.1.75)	184
unannotated gene in CAR600-02	RAST-annotated			Phage protein	118
unannotated gene in Ver097	RAST-annotated			FIG00450668 hypothetical protein	136
