# Supplementary material for: Mutation-Driven Divergence and Convergence Indicate Adaptive Evolution of the Intracellular Human-Restricted Pathogen, Bartonella bacilliformis
Source: PLoS Negl Trop Dis. 2016 May 11;10(5):e0004712. doi: 10.1371/journal.pntd.0004712 (PMC4864206; doi:10.1371/journal.pntd.0004712)
Supplement: S4 Table — Gene annotations are based on the reference strain KC583. (PDF) [file pntd.0004712.s008.pdf]

S4 Table. List of genes with truncation mutations in one or multiple strains. Gene annotations are based on the reference strain KC583.

Gene	GI	Strand	CDS-region	Product	Protein length (AA)	Strain(s) accumulating truncation mutations
BARBAKC583_0008	120613918	-	7946-8785	conserved hypothetical protein	279	Ver097
ftsY	120614878	-	17965-19128	signal recognition particle-docking protein FtsY	387	CAR,HeidiMejia,Hosp
BARBAKC583_0239	120614207	+	238814-239101	conserved hypothetical protein	95	CAR,HeidiMejia,Hosp,VAB9028,Cond
BARBAKC583_0249	120614530	-	243884-244912	sensor histidine kinase	342	Ver097
BARBAKC583_0300	120614190	+	288857-289600	conserved hypothetical protein TIGR00046	247	COND044,
BARBAKC583_0304	120614186	+	292192-292308	hypothetical protein	38	Ver097
glyQ	120614424	-	379558-380508	glycyl-tRNA synthetase, alpha subunit	316	COND044, Ver097
rnhB	120613913	+	386884-387552	ribonuclease HII	222	Ver097
BARBAKC583_0403	120614974	-	402896-404314	TldD/PmbA family protein	472	CUSCO5, Peru-18
ppdK	120614145	+	419941-422628	pyruvate, phosphate dikinase	895	CUSCO5, Peru-18, Peru38, Ver075, COND044,
hmuU	120614828	-	460855-461955	hemin ABC transporter, permease protein	366	CAR, HeidiMejia, Hosp, VAB9028COND044, Ver097
gid	120614297	-	563823-565217	gid protein	464	Ver097
tig	120614827	+	565640-567064	trigger factor	474	CAR, HeidiMejia, Hosp, VAB9028COND044,
BARBAKC583_0573	120614065	+	586776-587462	nitroreductase family protein	228	Ver097
hflX	120614549	+	645286-646659	GTP-binding proten HflX	457	Ver097
plsX	120614449	+	655867-656937	fatty acid/phospholipid synthesis protein PlsX	356	CUSCO5, Peru-18, VAB9028
BARBAKC583_0757	120614385	-	771789-773549	putative microcin immunity protein	586	CAR, HeidiMejia, Hosp, VAB9028
accC	120614354	-	776689-778029	acetyl-CoA carboxylase, biotin carboxylase	446	Ver097
nuoE	120614336	+	792302-792979	NADH dehydrogenase (quinone), E subunit	225	Ver097
prfB	120614582	+	j824125-825256	peptide chain release factor 2	376	KC583, CAR, CUSCO5, HeidiMejia, Hosp, INS, Peru-18, Peru38, SanPedro, VAB9028, Ver075, COND044, Ver097
BARBAKC583_0834	120613931	-	852038-852577	cold-shock DNA-binding family protein	179	Ver097
dprA	120614558	+	860256-861446	DNA protecting protein DprA	396	CAR, HeidiMejia, Hosp, VAB9028COND044
purL	120613874	+	882527-884740	phosphoribosylformylglycinamidine synthase II	737	COND044

BARBAKC583_0880	120615024	-	909304-910887	integral membrane protein TerC family/CBS/transporter domain	527	Ver097
murB	120613848	-	974816-975790	UDP-N-acetylenolpyruvoylglucosamine reductase	324	Ver097
BARBAKC583_1043	120614895	-	1079584-1080489	tetrapyrrole methylase family protein	301	Ver097
BARBAKC583_1124	120614670	-	1161608-1162180	transglycosylase, SLT domain protein	190	Ver097
motA	120614411	+	1189699-1190604	lateral flagellar motor protein MotA	301	Ver097
BARBAKC583_1264	120615000	+	1309992-1311389	cytosol aminopeptidase family protein	465	Ver097
irr	120614260	-	1313362-1313805	iron response regulator Irr	147	Ver097
recF	120614636	-	1321846-1322982	DNA replication and repair protein recF	378	Ver097
ccmC	120614889	+	1331364-1332143	heme exporter protein CcmC	259	Ver097
BARBAKC583_1318	120615063	-	1370518-1371690	rmuC domain protein	390	Ver097
BARBAKC583_1322	120614803	-	1374050-1374868	conserved hypothetical protein	272	Ver097
BARBAKC583_1339	120614861	-	1393752-1394483	conserved hypothetical protein	243	CAR, CUSCO5, HeidiMejia, Hosp, Peru-18, Peru38, VAB9028, COND044
folC	120614266	+	1415701-1417023	folylpolyglutamate synthase	440	CUSCO5, Peru-18, Ver075
BARBAKC583_1362	120614144	-	1435427-1436671	radical SAM enzyme, Cfr family	414	Ver097
unannotated gene in KC583	RAST-annotated			hypothetical protein	47	Ver097
unannotated gene in KC583	RAST-annotated			FIG00450845 hypothetical protein	83	COND044
unannotated gene in KC583	RAST-annotated			Peptide ABC transporter, periplasmic peptide-binding protein	43	COND044
unannotated gene in CAR600-02	RAST-annotated			RNA polymerase sigma factor RpoH-related protein	156	HeidiMejia, INS
unannotated gene in CAR600-02	RAST-annotated			hypothetical protein	46	Ver097
unannotated gene in CUSCO5	RAST-annotated			hypothetical protein	44	KC583, CARHeidiMejia, Hosp, INSPeru38, SanPedro, VAB9028, Ver075, COND044, Ver097
unannotated gene in CUSCO5	RAST-annotated			hypothetical protein	52	Ver097
unannotated gene in Peru18	RAST-annotated			hypothetical protein	48	COND044, Ver097
unannotated gene in CONDO44	RAST-annotated			hypothetical protein	39	CAR, HeidiMejia, Hosp, VAB9028,
unannotated gene in Ver097	RAST-annotated			hypothetical protein	38	KC583, CAR, CUSCO5, HeidiMejia, Hosp, INS, Peru-18, Peru38, SanPedro, VAB9028, Ver075, COND044

unannotated gene in Ver097	RAST-annotated	hypothetical protein	42	KC583, INS, Peru38, SanPedro, VAB9028, Ver075, COND044
unannotated gene in Ver097	RAST-annotated	hypothetical protein	46	KC583, CAR, CUSCO5, HeidiMejia, Hosp, INS, Peru-18, Peru38, SanPedro, VAB9028, Ver075, COND044
unannotated gene in Ver097	RAST-annotated	Putative autotransporter	91	KC583, CAR, CUSCO5, HeidiMejia, Hosp, INS, Peru-18, Peru38, SanPedro, VAB9028, Ver075, COND044
