# Supplementary material for: Mutation-Driven Divergence and Convergence Indicate Adaptive Evolution of the Intracellular Human-Restricted Pathogen, Bartonella bacilliformis
Source: PLoS Negl Trop Dis. 2016 May 11;10(5):e0004712. doi: 10.1371/journal.pntd.0004712 (PMC4864206; doi:10.1371/journal.pntd.0004712)
Supplement: S5 Table — Gene annotations are based on reference strain KC583. Amino acid positions accumulating convergent mutations are shown. Genes representing enriched (or overrepresented) functional clusters are grouped. (PDF) [file pntd.0004712.s009.pdf]

Table S5. List of candidate non-recombinant core genes with adaptive convergent mutations in *B. bacilliformis* subspecies I. Gene annotations are based on reference strain KC583. Amino acid positions accumulating convergent mutations are shown. Genes representing enriched (or overrepresented) functional clusters are grouped.

Gene	GI	Strand	CDS-region	Product	Protein length (AA)	Amino acid position(s) with convergent mutations	Enriched (overrepresented) functional clusters	Gene Ontology (GO) category	Genes detected other enriched clusters
recA	120615021	+	728971-730014	protein RecA	347	311	DNA repair	Biological Process	ATP-binding
addA	120614009	+	1425958-1429425	double-strand break repair helicase AddA	1155	211			ATP-binding
ruvA	120614122	-	162493-163146	Holliday junction DNA helicase RuvA	217	160, 216			ATP-binding
ligA	120614712	-	963536-965689	DNA ligase, NAD-dependent	717	284, 417, 484, 488, 589			Ligase
uvrC	120614432	-	524104-526080	excinuclease ABC, C subunit	658	497			
ruvC	120614840	-	163151-163654	crossover junction endodeoxyribonuclease RuvC	167	50			
mutM	120614315	-	1266148-1267023	formamidopyrimidine-DNA glycosylase	291	19, 82, 89, 142			
pgk	120614458	+	152911-154113	phosphoglycerate kinase	400	32, 34, 127, 325	Glucose metabolic process		ATP-binding
BARBAKC583_0106	120614835	-	120668-121321	Transaldolase	217	101			
pgi	120614854	+	297059-298708	glucose-6-phosphate isomerase	549	369, 406, 420			
eno	120614294	+	540998-542269	phosphopyruvate hydratase	423	252			
sucA	120613962	+	26939-29938	2-oxoglutarate dehydrogenase, E1 component	999	388			
gap	120614284	+	151605-152612	glyceraldehyde-3-phosphate dehydrogenase, type I	335	72, 80			
topA	120613875	+	861827-864436	DNA topoisomerase I	869	714	ATP-binding	Molecular Function	
proB	120614801	+	311166-312305	glutamate 5-kinase	379	68			
BARBAKC583_0595	120615055	-	609473-610252	amino acid ABC transporter, ATP-binding protein	259	129			
BARBAKC583_0874	120613938	-	901154-902569	DEAD/DEAH box helicase domain/helicase conserved C-terminal domain protein	471	87, 402			
accD	120614862	+	1414777-1415697	acetyl-CoA carboxylase, carboxyl transferase, beta subunit	306	193			Ligase
pyrG	120614281	+	538394-540022	CTP synthase	542	164			Ligase
guaA	120614786	+	338944-340500	GMP synthase	518	43			Ligase
murD	120614097	-	979588-980994	UDP-N-acetylmuramoylalanine--D-glutamate ligase	468	448			Ligase

BARBAKC583_1007	120614488	+	1041651-1042682	ATPase, AAA family	343	306			
atpD	120614434	+	128216-129829	ATP synthase F1, beta subunit	537	507			
ndvA	120614225	-	732964-734751	beta-(1--2)glucan export ATP-binding protein	595	593			
glyS	120614808	-	377343-379565	glycyl-tRNA synthetase, beta subunit	740	477			Ligase
folC	120614266	+	1415701-1417023	folylpolyglutamate synthase	440	3, 339	Ligase		
cobT	120613980	-	88851-90746	cobaltochelatase, CobT subunit	631	302, 594			
coaBC	120613971	+	1412156-1413391	phosphopantothenoylcysteine decarboxylase/phosphopantothenate--cysteine ligase	411	136, 301			
rplQ	120613826	+	725185-725610	ribosomal protein L17	141	126			
ybgC	120615028	-	160943-161383	tol-pal system-associated acyl-CoA thioesterase	146	142			
BARBAKC583_0153	120614495	-	164316-164834	conserved hypothetical protein	172	172			
BARBAKC583_0651	120613855	+	668413-669087	putative protein-L-isoaspartate O-methyltransferase	224	189			
BARBAKC583_1364	120614456	-	1436699-1437208	conserved hypothetical protein	169	13			
aroK	120614783	-	43873-44418	shikimate kinase	181	79			
BARBAKC583_0099	120613956	-	112169-113143	FtsX family protein	324	262			
BARBAKC583_0335	120614924	+	329234-330226	sugar isomerase, KpsF/GutQ family	330	155			
atpG	120614109	+	127285-128193	ATP synthase F1, gamma subunit	302	207			
BARBAKC583_0866	120614184	+	885545-886798	drug resistance transporter, Bcr/CflA subfamily	417	350			
BARBAKC583_0763	120614179	-	778743-779540	thioredoxin domain protein	265	253			
BARBAKC583_0064	120614476	+	68364-69959	conserved hypothetical protein	531	458			
fliF	120614922	-	1166740-1168401	flagellar M-ring protein FliF	553	7			
BARBAKC583_1355	120614865	+	1421150-1422643	P-loop hydrolase/phosphotransferase	497	494			
BARBAKC583_0816	120614164	-	835542-836756	ubiquinone biosynthesis hydroxylase, UbiH/UbiF/VisC/COQ6 family	404	262			
BARBAKC583_0623	120614724	-	639489-644120	conserved hypothetical protein	1543	290, 372, 400, 972			
phoU	120614136	-	1269493-1270215	phosphate transport system regulatory protein PhoU	240	74			
frr	120614546	+	599104-599664	ribosome recycling factor	186	4			
BARBAKC583_1340	120614452	-	1394539-1395318	conserved hypothetical protein	259	182			
rpsF	120614035	-	500114-500524	ribosomal protein S6	136	25			
BARBAKC583_1097	120614334	+	1129814-1130476	ATP-dependent protease	220	145			

nspC	120614968	-	34049-35146	carboxynorspermidine decarboxylase	365	310			
BARBAKC583_0996	120614293	+	1032588-1033379	conserved hypothetical protein TIGR00726	263	262			
BARBAKC583_0230	120614233	+	230650-231825	conserved hypothetical protein	391	310			
nuoM	120614200	+	801150-802619	NADH dehydrogenase (quinone), M subunit	489	327			
nuoD	120614091	+	791112-792302	NADH dehydrogenase (quinone), D subunit	396	286			
BARBAKC583_1090	120614729	-	1118676-1119809	pyridoxal-dependent decarboxylase	377	77			
BARBAKC583_0895	120614363	+	921960-923168	transporter, major facilitator family	402	277			
BARBAKC583_0305	120614903	+	292600-293736	efflux transporter, RND family, MFP subunit	378	92			
mraY	120613942	-	980999-982069	phospho-N-acetylmuramoyl-pentapeptide- transferase	356	87			
BARBAKC583_0094	120614660	+	105736-106368	LolA family protein	210	40			
BARBAKC583_0408	120614976	-	408419-409324	conserved hypothetical protein	301	258, 288			
gcvP	120614195	-	1124711-1127506	glycine dehydrogenase	931	299, 448			
BARBAKC583_0096	120614847	+	107648-108847	putative macrolide-specific efflux protein MacA	399	16			
lys1	120614193	-	32755-33993	saccharopine dehydrogenase	412	204			
BARBAKC583_1220	120613893	-	1264659-1266074	peptidase, M20/M25/M40 family	471	100			
BARBAKC583_0178	120615003	-	187140-187673	deoxyuridine 5'-triphosphate nucleotidohydrolase	177	163			
BARBAKC583_0743	120614577	-	752211-753425	isocitrate dehydrogenase, NADP-dependent	404	404			
BARBAKC583_0904	120614211	-	932336-933100	N-acetylmuramoyl-L-alanine amidase family protein	254	41, 107, 248			
